# Highly sensitive electrochemical detection of cholesterol based on Au–Pt NPs/PAMAM–ZIF-67 nanomaterials

**DOI:** 10.1007/s44211-023-00427-0

**Published:** 2023-09-25

**Authors:** Liangtian Zhang, Jianmeng Zhu, Wenzhong Hong, Gang Li

**Affiliations:** 1 Emergency and Intensive Care Unit Center, Department of Intensive Care Unit, Chun’an First People’s Hospital, Zhejiang Provincial People’s Hospital Chun’an Branch, Hangzhou Medical College Affiliated Chun’an Hospital, Hangzhou, Zhejiang People’s Republic of China; 2 Clinical Laboratory of Chun’an First People’s Hospital, Zhejiang Provincial People’s Hospital Chun’an Branch, Hangzhou Medical College Affiliated Chun’an Hospital, Hangzhou, Zhejiang People’s Republic of China; 3 Department of Emergency Medicine, The Third Affiliated Hospital of Zhejiang Chinese Medical University, Hangzhou, 310000 Zhejiang People’s Republic of China

**Keywords:** Cholesterol, Biosensor, Zeolitic imidazolate frameworks, Nanoparticles

## Abstract

A cholesterol biosensor was constructed by bimetallic (Au and Pt) and poly(amidoamine)-zeolite imidazole framework (PAMAM–ZIF-67). First, PAMAM–ZIF-67 nanomaterial was immobilized onto the electrode, and then Au and Pt were modified on the electrode by the electro-deposition method. Subsequently, cholesterol oxidase (ChOx) and cholesterol esterase (ChEt) were fixed on the electrode. The stepwise modification procedures were recorded by impedance spectroscopy and voltammetry. The current response presented a linear relation to the logarithm of cholesterol content when content ranged between 0.00015 and 10.24 mM, and the minimum detection concentration reached 3 nM. The electrode was also used for the cholesterol assay in serum, which hinted at its potentially valuable in clinical diagnostics.

**Graphical abstract:**

An electrochemical biosensor based on gold nanoparticles, platinum nanoparticles, and polyamide–zeolitic imidazolate frameworks was developed for detection of cholesterol. First, polyamide–zeolitic imidazolate frameworks nanomaterial was fixed onto the electrode modified of mercaptopropionic acid by Au–S bond. Then, gold nanoparticles and platinum nanoparticles were electrodeposited on the above electrode. Subsequently, cholesterol oxidase and cholesterol esterase were co-immobilized on the surface of the modified electrode to fabricate the cholesterol biosensor. The biosensor has also been used for the measurement of cholesterol in human serum, which implied potential applications in biotechnology and clinical diagnostics.
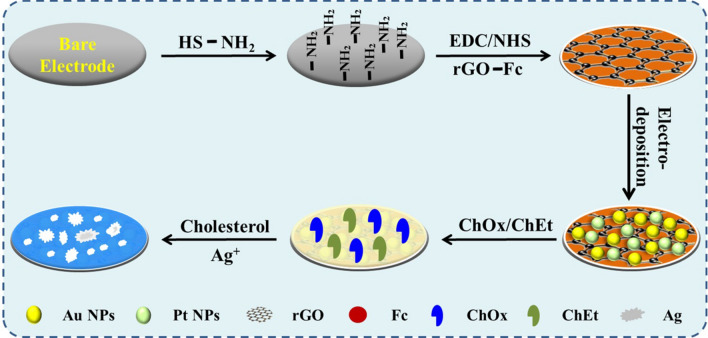

## Introduction

Cholesterol is an indispensable lipid for biological tissue cells and essential for forming steroid hormones and bile acids [1, 2]. It is produced by the liver and widely exists in nerve tissue, having a significant effect for the people [3]. Cholesterol, normal levels in human serum from 120 to 260 mg/dl [4, 5], is also an important indicator of clinical biochemical tests [6, 7]. High content cholesterol accumulation may lead to arteriosclerosis and lipid metabolism dysfunction [8, 9]. Therefore, constant cholesterol monitoring is necessary for medical diagnosis [10, 11]. Some methods have been reported and used for cholesterol measurement, containing high-performance liquid chromatography, spectrophotometric and other methods [12]. Because of its excellent properties, electrochemical biosensors have attracted lots of attention [13, 14].

Electrochemical biosensors are composed of the sensing element and conversion element. The current response signal from the electronic transport chain occurs between the electrode and embellished materials. With the development of research, cholesterol oxidase (ChOx) is widely applied in the medical field as a sensing element [15, 16], which could catalyze cholesterol into an intermediate of cholest-4-en-3-one and H_2_O_2_. Simultaneously, the Ag^+^ in glycine solution is reduced into metallic Ag by the produced H_2_O_2_, and that response current can be quickly detected by the developed biosensor [17, 18]. Overall, a biosensor monitoring enzymatically produced H_2_O_2_ is necessary to catch the H_2_O_2_ accurately.

Metal–organic frameworks (MOFs) are composed of organic ligands and inorganic metals [19, 20] and have the characteristics of large surface area, widely used in the fields of electro-catalysis, sensing, and sewage treatment [21]. Among them, zeolitic imidazolate frameworks (ZIFs) are a class of MOFs with zeolite topological structures [22]. Gao et al. use ZIFs-67 as the carrier to construct a nano-drug delivery system CaO_2_@Adriamycin@ZIF-67 for tumor chemodynamic therapy [23]. Li et al. prepared Glucose Oxidase/Horseradish Peroxidase-loaded ZIF-67@CaO_2_ to detect squamous cell carcinomas' biomarkers [24].

Metal nanoparticles have important applications in electrochemical biosensors; they are excellent catalysts that increase the electrochemical reaction rate, and they can still be used as conductors to increase conductivity from catalytic reaction centers of the enzyme to electrode surfaces [25, 26]. Bai et al. constructed the platinum particles-modified electrode to detect H_2_O_2_ in living cells, exhibiting outstanding catalytic capability for H_2_O_2_ [27]. Zhang et al. developed a cholesterol sensor based on gold nanoparticle catalysis and showed excellent properties for detecting cholesterol [28].

In this paper, we fabricated a cholesterol sensor based on bimetal nanoparticles and poly(amidoamine)-zeolite imidazole framework (PAMAM–ZIF-67). The electrode was modified with the PAMAM–ZIF-67 nanomaterial and electrodeposited on Au and Pt. Then, cholesterol oxidase (ChOx) and cholesterol esterase (ChEt) were fixed on the electrode. Through enzyme cooperation and the synergic effect of PAMAM–ZIF-67 and bimetallic, current response of the modified electrodes can be detected by an electrochemical workstation. The biosensor exhibits an excellent current response, exhibiting appropriate detection performance.

## Experiment

### Reagents

*N*-(3-Dimethylaminopropyl)-*N*-ethyl-carbodiimide hydrochloride (EDC), ChEt, ChOx, and cholesterol were obtained from Sigma. Chloroauric acid (HAuCl_4_), chloroplatinic acid (H_2_PtCl_6_), mercaptoethylamine (MPA), Co (NO_3_)_2_·6H_2_O, ascorbic acid, estradiol, glucose, uric acid, and Triton X-100 were purchased from Sigma-Aldrich. Analytical-grade reagents were used in the test.

### Apparatus

The morphologies are photographed by scanning electron microscopy (JSM-6700). Current signal is performed on electrochemical workstation (CHI 660). The conventional three-electrode system is used in the tests. A saturated calomel electrode (SCE) stands for the reference electrode, a platinum wire stands for the auxiliary electrode, and ChOx&ChEt/Au–Pt NPs/PAMAM–ZIF-67 electrode stands for the working electrode. Linear sweep voltammetry (LSV) was carried out in KNO_3_ solution (0.6 M). Electrochemical impedance spectroscopy (EIS) and cyclic voltammetry (CV) were executed in a [Fe(CN)_6_]^3−/4−^ solution.

### Composition of PAMAM–ZIF-67 nanomaterial

The 2-methylimidazole (26 mmol) and Co(NO_3_)_2_•6H_2_O (3 mmol) were mixed in methanol (90 ml) to prepare ZIF-67 [29, 30]. The mixture was centrifuged, washed, and dried for subsequent use.

Take 2.2 ml of ethylenediamine in a 100 ml three-necked flask, add 8.74 g of methanol as a solvent, place it in an ice water bath under the protection of nitrogen, and add 24.0 ml of methyl acrylate dropwise, with the dropping rate controlled at one drop per second. Continue to stir for 24 min. Purify the product; use the above product and excess ethylenediamine to carry out the phthalimide reaction, and alternately repeat the above steps to obtain the PAMAM dendrimer [31]. ZIF-67 (1 mL, 1.0 mg/mL) and PAMAM (1 mL, 0.5 mg/mL) were mixed for 12 h and then centrifuged and washed. The prepared PAMAM–ZIF-67 was re-dispersed in double-distilled water for the next experiment.

### Construction of biosensor

The bare electrode was buffed with 0.05 µm aluminum oxide and washed for 5 min in ethanol and H_2_O. The electrode was immersed in MPA (20 μl 10 μM) solution at 4 °C and washed with PBS after 12 h Subsequently, the electrode was immersed in an EDC/NHS solution (10 mM) for 0.5 h and then cleaned with PBS. The PAMAM–ZIF-67 solutions were dropped on the prepared electrode and kept for 1 h. The electrode was rinsed with 0.1 M PBS and then dipped into 5 mL 0.01% HAuCl_4_ solution. Au nanoparticles were deposited by amperometry at − 0.5 V. Similarly, the electrode was rinsed with 0.1 M PBS and then dipped into 5 mL 0.01% H_2_PtCl_6_ solution. Pt nanoparticles were deposited by amperometry at − 0.5 V. The electrode was soaked in EDC/NHS for 20 min; then, ChOx (10 μl 2 mg/ml) and ChEt (10 μl 2 mg/ml) were modified onto electrode and kept at 37 °C. Finally, the electrode was rinsed to remove unattached enzymes and the biosensor was successfully constructed. The modified electrodes were immersed in the AgNO_3_ solution with different content cholesterol. AgNO_3_ can be catalytic reduced to Ag elemental by H_2_O_2_, which is used as a detectable conversion electrical signal. In the detection of cholesterol, cholesterol is oxidized to cholest-4-en-3-one by the flavin cofactor. The reduced cofactor is recycled by oxygen to produce H_2_O_2_, and then the Ag^+^ in glycine solution are reduced into metallic Ag by H_2_O_2_. Detection of the enzymatically deposited Ag is achieved by anodic stripping voltammetry. Current responses were recorded by LSV in the KNO_3_ solution (scan potential: − 0.2 to 0.8 V; rate: 100 mV/s).

### Exploration of biosensor performance

To explore selectivity performance, lots of potential interfering materials were detected instead of cholesterol under the same conditions. The current caused by changes in silver content produced by enzyme catalysis was obtained.

### Statistical analysis

The dates are shown though the average value and SDs. For statistical review, a between-groups independent t-test was conducted. A significant difference was performed.

## Results and discussion

### Rationale of the sensor

**Fig. 1:**
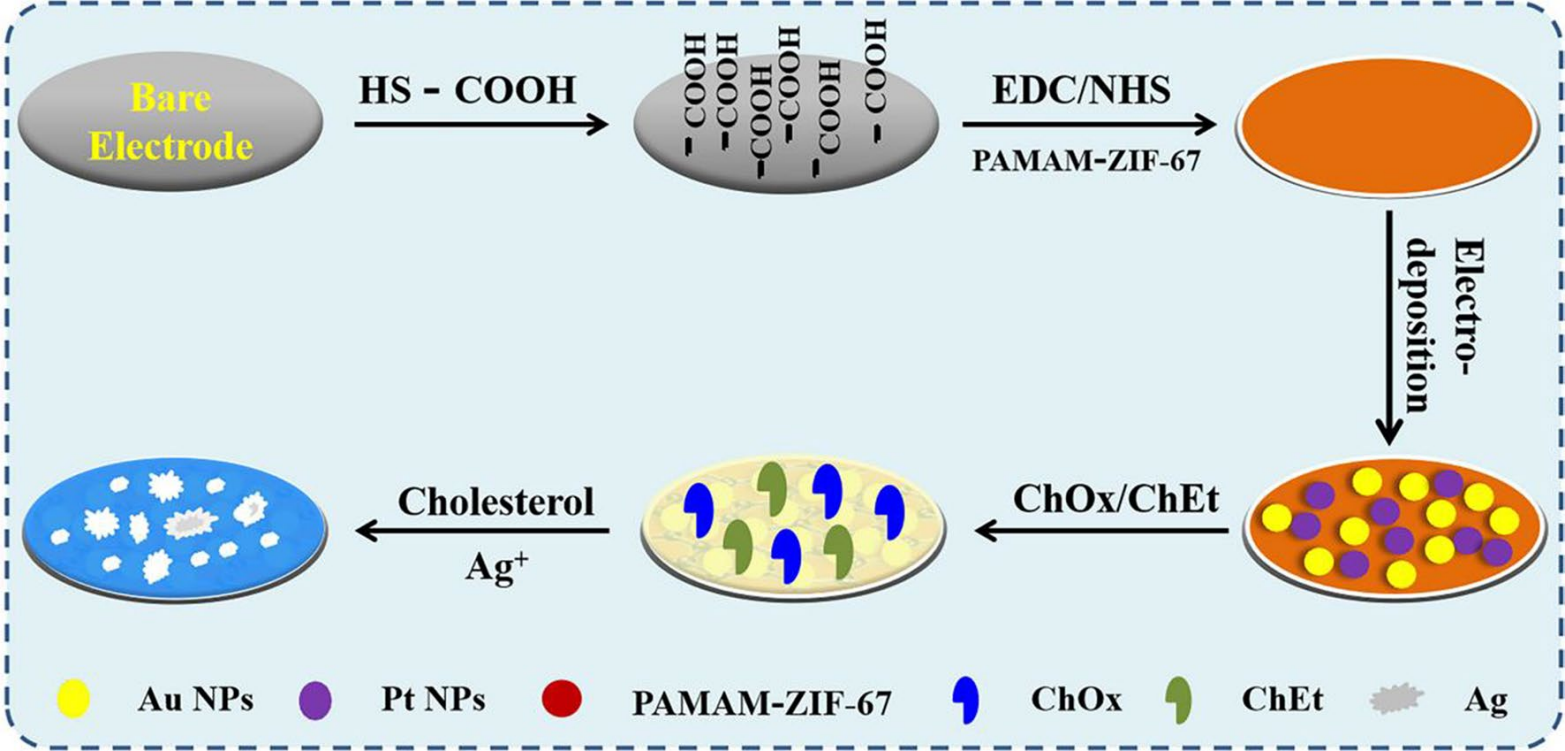
Principle of the ChOx&ChEt/Au–Pt NPs/PAMAM–ZIF-67 biosensor for the detection of cholesterol

The construction process of the electrode is schematically explicated in Fig. 1. PAMAM–ZIF-67 solution was immobilized on the electrode, and then Au and Pt nanoparticles were electrodeposited. Subsequently, enzymes were fixed on the electrode surface. Nafion solution (5 µl 0.5%) was injected on the electrode to eliminate foreign interferences and prevent possible enzyme leakage. The biosensor was constructed. ChOx, a flavin adenosine dinucleotide (FAD)-containing enzyme, could catalyze cholesterol to be H_2_O_2_ and cholest-4-en-3-one [27]. Subsequently, Ag^+^ in glycine solution was reduced to Ag by the generated H_2_O_2_. Detection of produced elemental silver was carried out through anodic stripping voltammetry (ASV). The modified electrode could be used for cholesterol detection.

### Characterization of PAMAM–ZIF-67 nanomaterial

**Fig. 2:**
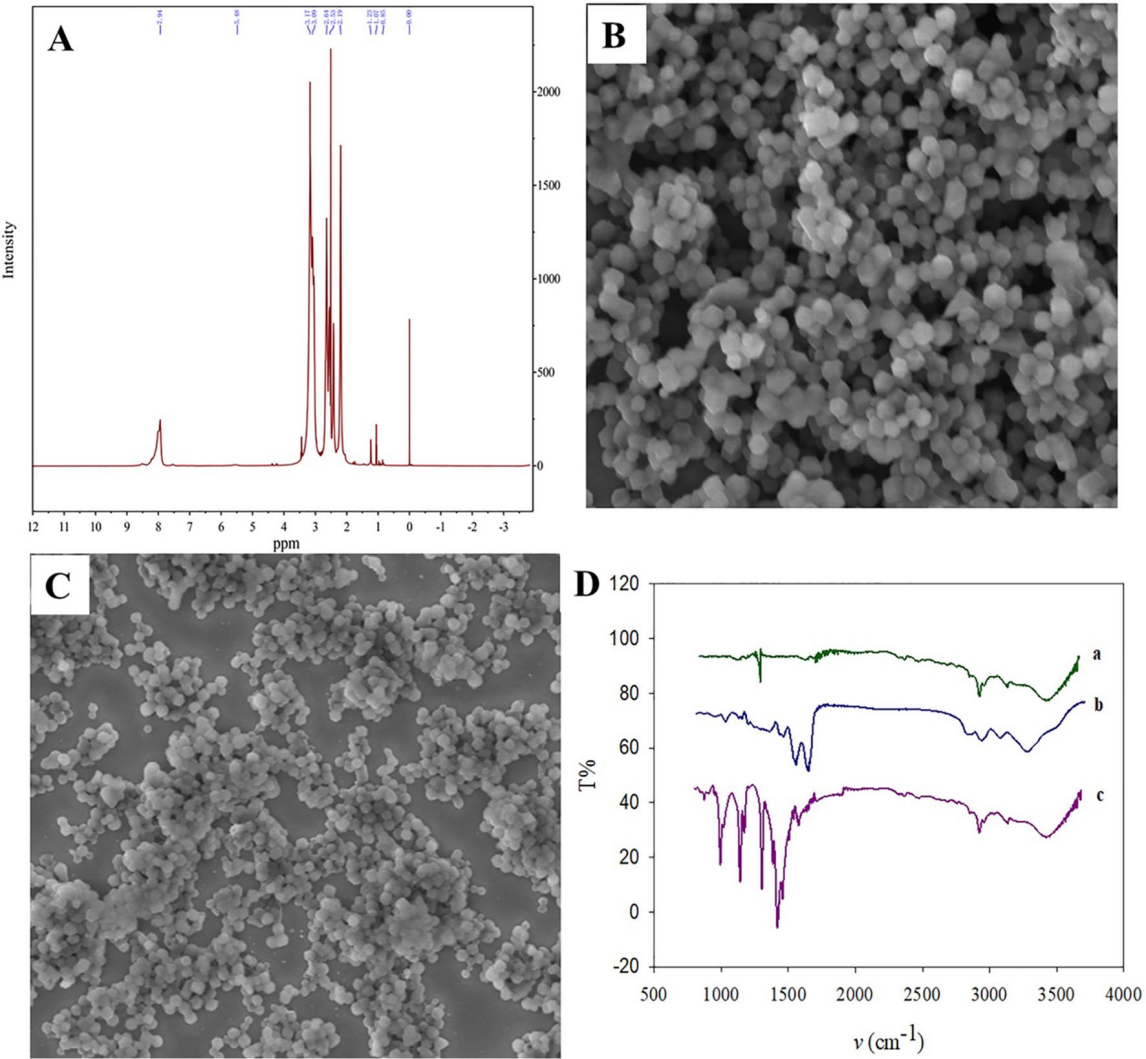
Characterization of PAMAM–ZIF-67. **A** ^1^H NMR spectra of PAMAM, **B** SEM image of ZIF-67, **C** SEM image of PAMAM–ZIF-67, **D** FT-IR spectra of (a) ZIF-67, (b) PAMAM, (c) PAMAM–ZIF-67

ZIF-67–PAMAM was characterized with SEM, NMR, and FT-IR. In the ^1^H NMR spectra, the peaks at 2.19, 2.53 and 2.64 ppm can be the characteristic of PAMAM of NH_2_, CH_2_CONH, and NCH_2_, respectively (Fig. 2A). The SEM photograph of ZIF-67 presents the spherical structure and the average size of spherical was about 100 nm (Fig. 2B). The SEM photograph of PAMAM–ZIF-67 is presented in Fig. 2C, the sizes of spherical particles are about 100 nm. Figure 2D shows the infrared spectrogram of ZIF-67 (trace a), PAMAM (trace b), and PAMAM–ZIF-67 (trace c). In the infrared spectrogram of ZIF-67, the 1280 cm^−1^ peak is the bending vibration of the imidazole ring. In the infrared spectrogram of PAMAM, the 3252 cm^−1^ peak represents the stretching vibration of –NH_2_, and the peak 1547 cm^−1^ is attributed to the stretching vibration of the C–N bond. The peaks at 3520 cm^−1^ and 1662 cm^−1^ are the stretching vibrations of OH and C=C, respectively. In the infrared spectrogram of PAMAM–ZIF-67, the 1580 cm^−1^ peak represents the C=N stretching vibration of 2-methylimidazole, the 1430 cm^−1^ and 1216 cm^−1^ are attributed to the stretching and bending vibration of the imidazole ring.

### Electrochemistry characteristics of the sensor

**Fig. 3:**
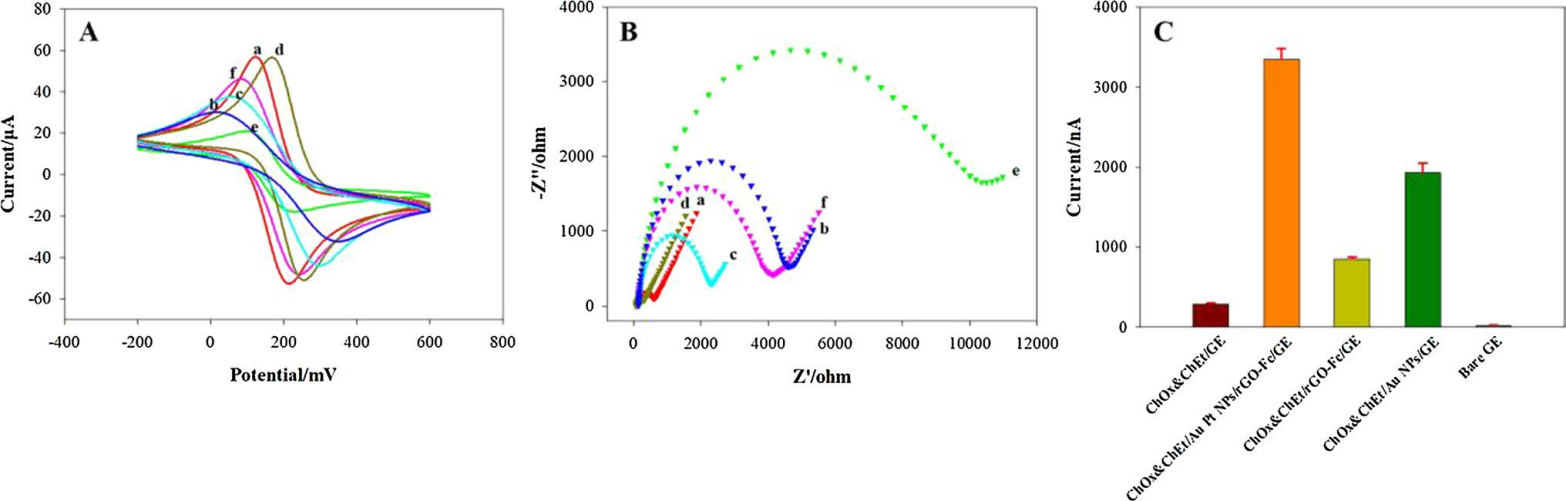
Electrochemical behaviors of the biosensor. **A** CV response of (red curve a) bare GE, (blue curve b) MPA/GE, (cyan curve c) PAMAM–ZIF-67/MPA/GE, (brown curve d) Au–Pt NPs/PAMAM–ZIF-67/MPA/GE, (green curve e) ChOx&ChEt/Au–Pt NPs/PAMAM–ZIF-67/MPA/GE, (pink curve f) Ag/ChOx&ChEt/Au–Pt NPs/PAMAM–ZIF-67/MPA/GE in an [Fe(CN)_6_]^3−/4−^ solution from -0.4 to 0.8 V with a 100 mV/s scanning rate. **B** EIS of (red curve a) bare GE, (blue curve b) MPA/GE, (cyan curve c) PAMAM–ZIF-67/MPA/GE, (brown curve d) Au–Pt NPs/PAMAM–ZIF-67/MPA/GE, (green curve e) ChOx&ChEt/Au–Pt NPs/PAMAM–ZIF-67/MPA/GE, (pink curve f) Ag/ChOx&ChEt/Au–Pt NPs/PAMAM–ZIF-67/MPA/GE in PBS containing 5 mM Fe(CN)_6_^3−^/^4−^ at 0.24 V (versus SCE) with a frequency range of 0.1–100 kHz. **C** Current response with the different modifications, including ChOx&ChEt/Au–Pt NPs/PAMAM–ZIF-67/GE, ChOx&ChEt/PAMAM–ZIF-67/GE, ChOx&ChEt/Au NPs/GE, bare GE and ChOx&ChEt/GE. (The error bars represent the standard error of the mean (*n* = 3 electrodes)

The electrochemistry performance of the modified electrode was studied by CV. Figure 3A shows the response current change of electrodes in the K_4_Fe(CN)_6_/K_3_Fe(CN)_6_–0.1 mol/L KCl solution (5 mmol/L). The unmodified electrode shows quasi-reversible redox peaks (red line a). When MPA was fixed on the surface of a bare electrode, the current decreased sharply (blue line b), and when the PAMAM–ZIF-67 was cast on MPA/GE, a current response was gained (cyan line c). After Au and Pt nanoparticles were electrodeposited on the above electrode, current response further increased, as Au and Pt NPs are analogous to a conductor (brown line d). When the enzymes were fixed on the electrode surface, the current weakened sharply (green line e), due to the poor electro-conductibility. After the enzymatic silver deposition was developed, the signal was gained (pink line f) because of silver's extremely good electron transfer performance.

EIS has been widely used to investigate the various processes of electrode modification with electron transfer kinetics. Figure 3B presents the EIS results of electrodes. The resistance value of bare GE was 247 Ω (red line a). When mercaptopropionic acid was modified on the bare electrode, the impedance value reached 4610 Ω (blue line b). The PAMAM–ZIF-67 was immobilized upon the MPA/GE, and the impedance value decreased to 2310 Ω (cyan line c). As the Au and Pt nanoparticles were deposited, the impedance value was 215 Ω (brown line d). When the enzyme was absorbed on the Au–Pt/ZIF-67–PAMAM electrode, the impedance value achieved to 12,650 Ω, owing to the weak conductivity (green line e). After the silver substance was deposited on the electrode surface, the impedance value of the electrode becomes dropped to 3740 Ω (pink line f), which attribute to the excellent electron transfer performance of silver.

Figure 3C displays the response signal of the modified electrode for the cholesterol test. When a certain amount of cholesterol was injected into the Ag^+^ solution, the modified electrodes exhibited different current signals. Compared to the other electrodes, containing bare electrode, ChOx&ChEt/PAMAM–ZIF-67-modified electrode, ChOx&ChEt/Au–Pt-modified electrode, and ChOx&ChEt-modified electrode, ChOx&ChEt/Au–Pt NPs/PAMAM–ZIF-67-modified electrode generated a significantly stronger response signal.

### Characterization of electrode

**Fig. 4:**
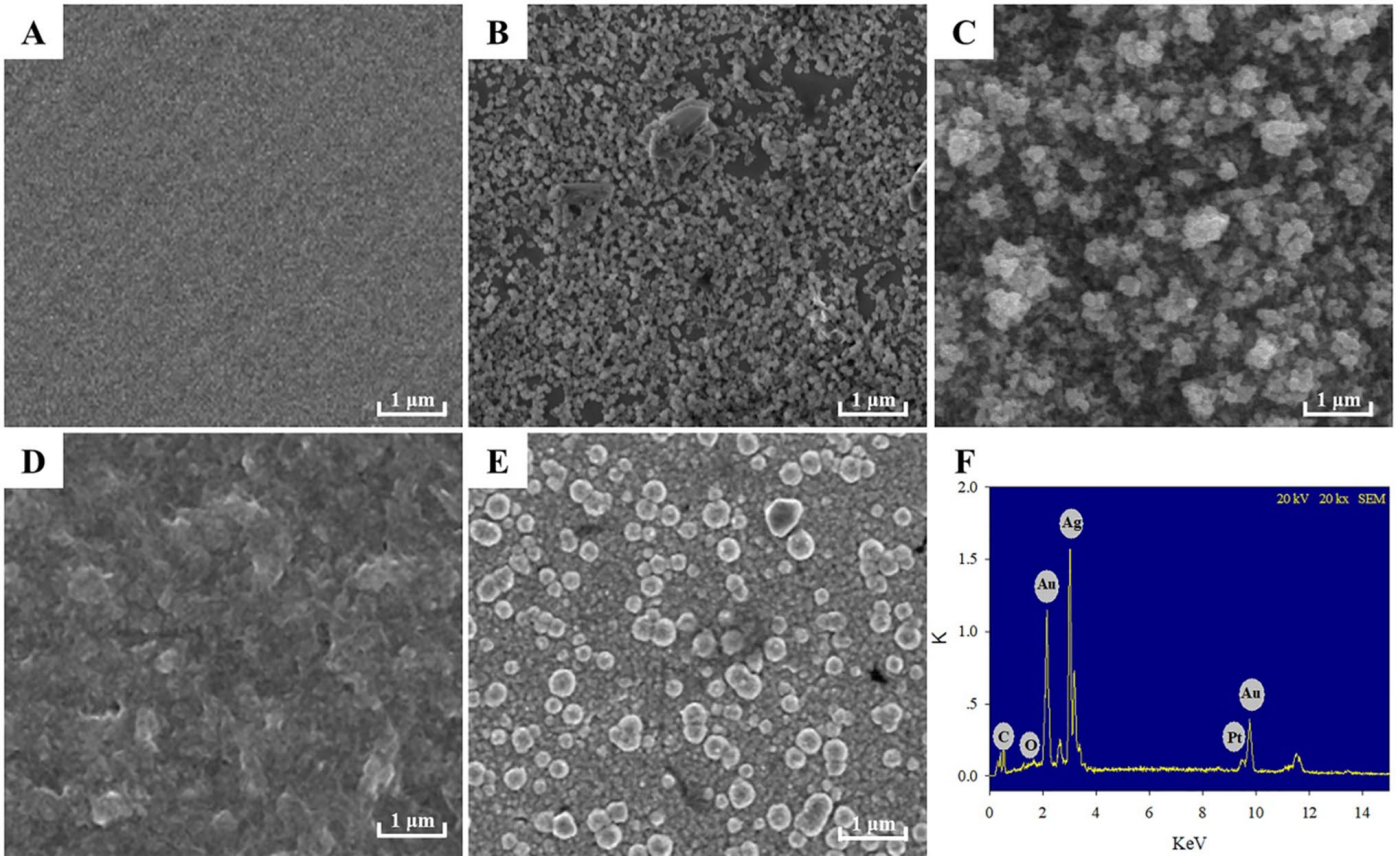
SEM images of **A** the bare electrode, **B** PAMAM–ZIF-67/GE, **C** Au–Pt NPs/PAMAM–ZIF-67/GE, **D** ChOx&ChEt/Au–Pt NPs/PAMAM–ZIF-67/GE, **E** Ag/ChOx&ChEt/Au–Pt NPs/PAMAM–ZIF-67/GE, **F** EDS images of Ag/ChOx&ChEt/Au–Pt NPs/PAMAM–ZIF-67/GE

Figure 4 reveals an SEM photograph of the biosensor fabrication procedure. The images of the bare electrode show uniform darkness (Fig. 4A). The modified electrode exhibited a spherical morphology when PAMAM–ZIF-67 was immobilized (Fig. 4B). Along with the electrodeposited Au and Pt nanoparticles, many bright particles were randomly observed on the surface of PAMAM–ZIF-67/GE (Fig. 4C), which implied that the Au and Pt nanoparticles had been successfully deposited on the electrode. Subsequently, Au and Pt nanoparticles disappeared and were covered by dense ChOx and ChEt enzymes, as shown in Fig. 4D. When cholesterol was added to the electrolyte solution, it degraded and generated hydrogen peroxide on the surface of the electrode, and then silver ions were reduced to silver by hydrogen peroxide. Therefore, lots of white silver flakes were deposited on the modified electrode surface (Fig. 4E). The generated Ag was detected by energy-dispersive spectrometry (EDS), where Ag was exclusively observed with high intensity (Fig. 4F).

### Optimize assay parameters

**Fig. 5:**
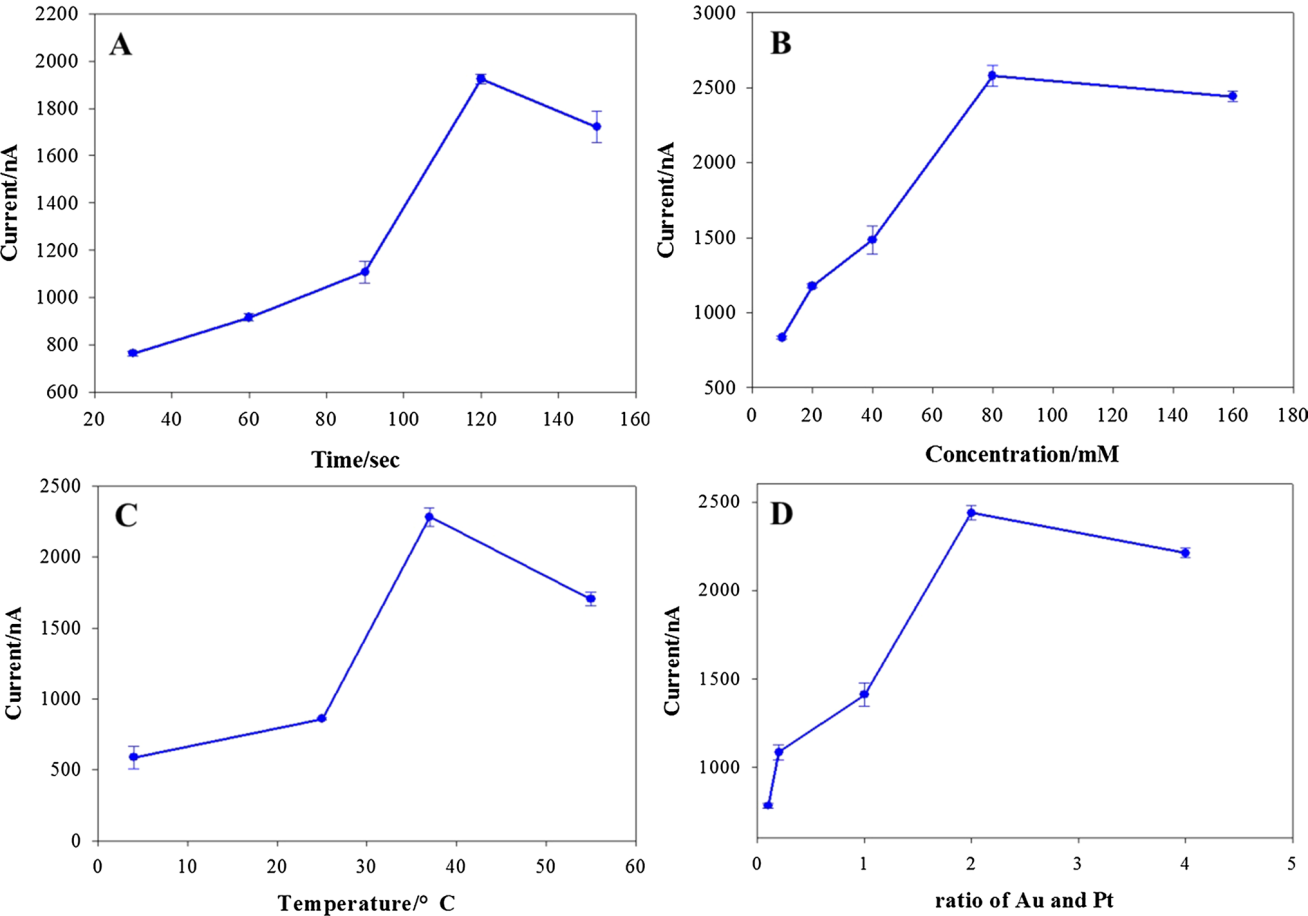
**A** Effect of the electro-deposition time of Au–Pt NPs on the electrode. **B** Optimization of the concentration of Ag^+^ in the glycine buffer. **C** Optimization of the temperature of the deposition reaction on the electrode. **D** Effect of the ratio of Au and Pt on the current response. The current was recorded using LSV measurements in 0.1 M HNO_3_ solution containing 0.6 M KNO_3_. The error bars are the standard error of the mean (*n* = 3 electrodes)

To ameliorate biosensor performance, some assay parameters were optimized, which contain the deposition time of the nanoparticles, the ratio of gold to platinum, Ag^+^ content, and enzymatic deposition conditions. The effect of the different deposition times of metal nanoparticles was investigated (Fig. 5A). The response current signal increased over the deposition time and climaxed at 120 s; a further increase in the deposition time caused the signal to decline slightly. This phenomenon is because as deposition occurs, the metal particles gradually gather as the specific surface area increases. As the time further increases, the metal particles aggregate and progressively form a sheet-like structure, reducing the specific surface area and weakening surface energy. All of these factors decrease the catalytic performance of the metal nanoparticles and the ability to load the enzyme. Thus, 120 s was selected for subsequent tests.

Increasing the Ag^+^ concentration could enhance the peak current due to the generated silver. Upon further improved the contents, the response signal declined (Fig. 5B). Therefore, 80 mM Ag^+^ was selected for further experiments.

As shown in Fig. 5C, enzymatic deposition occurred during the process of cholesterol disintegration into hydrogen peroxide, which affected the sensitivity of the biosensor. Thus, the temperature and time of enzymatic deposition were optimized for further detection. When the temperature increased from 4 to 37 °C, the generated signal raised. Higher temperatures decreased the enzyme activity and reduced the generation of hydrogen peroxide, which prevented the Ag^+^ in the solution from being reduced substantially and decreased the current response. So, 37 °C was used for enzymatic deposition.

Moreover, in linear sweep voltammetry, the ratio of Au to Pt was another factor affecting the electrochemical response signal. As the ratio increased from 1:10 to 2:1, the current response increased. When it was 4:1, the current appeared to drop slightly. Therefore, a ratio of 2:1 was selected for further experiments (Fig. 5D).

### Properties of the modified electrode

**Fig. 6:**
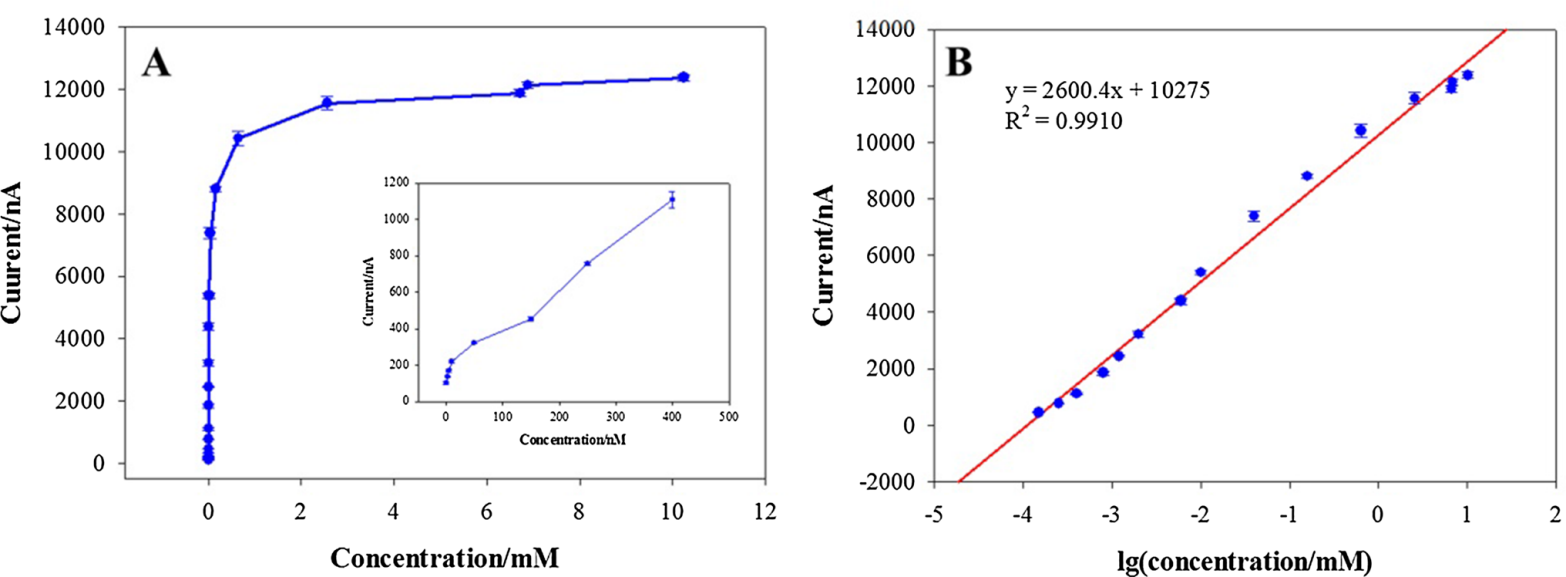
**A** Current response of the ChOx&ChEt/Au–Pt NPs/PAMAM–ZIF-67 biosensor toward cholesterol ranging from 0.3 × 10^−5^ to 10.24 mM. The concentration of cholesterol (0–400 nM) is given in the inset in (**A**). **B** Linear relation between current response and logarithm of cholesterol concentration. The error bars are the standard error of the mean (*n* = 3 electrodes)

Under optimal parameters, the electrode performance was assessed by linear sweep voltammograms. Figure 6A exhibits the biosensor response signal toward different cholesterol content (0.00005–10.24 mM). Figure 6B displays a linear correlation with content cholesterol in 0.00015–10.24 mM (*R*^2^ = 0.9910). The minimum detection concentration of 3 nM was gained under the signal-to-noise ratio of 3, and the concentration of cholesterol (0–400 nM) is given in the inset. A comparison of cholesterol biosensor-modified electrodes with different materials is presented. As shown in Table 1, the biosensor exhibited better performance toward cholesterol.

**Table 1:** Comparison of cholesterol biosensor-modified electrodes

Nanomaterial	Sensing element	Linear range (mM)	LOD (μM)	References
Au Nanowire	ChOx, ChEt	0.01–0.06		[32]
SnO_2_ NPs	ChOx	0.26–10.36	130	[33]
ZnO–CuO/ITO	ChOx, ChEt	0.5–12	500	[3]
G/Ti(G)-3DNS/CS	ChOx	0.05–8.0	6	[5]
Cu_2_O NPs	ChOx, ChEt	0.259–11.64		[34]
AuNPs–MWCNTs	ChOx	0.01–5.0	4.3	[35]
PSBTz/β-CD	ChOx	150–22,500		[36]
Graphene oxide	ChOx	0.0005–0.0465		[37]
Au–Pt NPs/PAMAM–ZIF-67	ChOx, ChEt	0.00015–10.24	0.003	This work

### Stability, selectivity, and reproducibility study

The modified electrodes were measured repeatedly to investigate the electrode stability. After the 8th measurement, the signal of the modified electrode remained at approximately 90% of its original signal.

Under the same conditions, six prepared electrodes were used to detect cholesterol content, and the reproducibility of the biosensor was tested by the generated current signal. The relative standard deviation (RSD) value reached 4.2%. The dates showed that the modified electrodes possess feasible reproducibility.

**Fig. 7:**
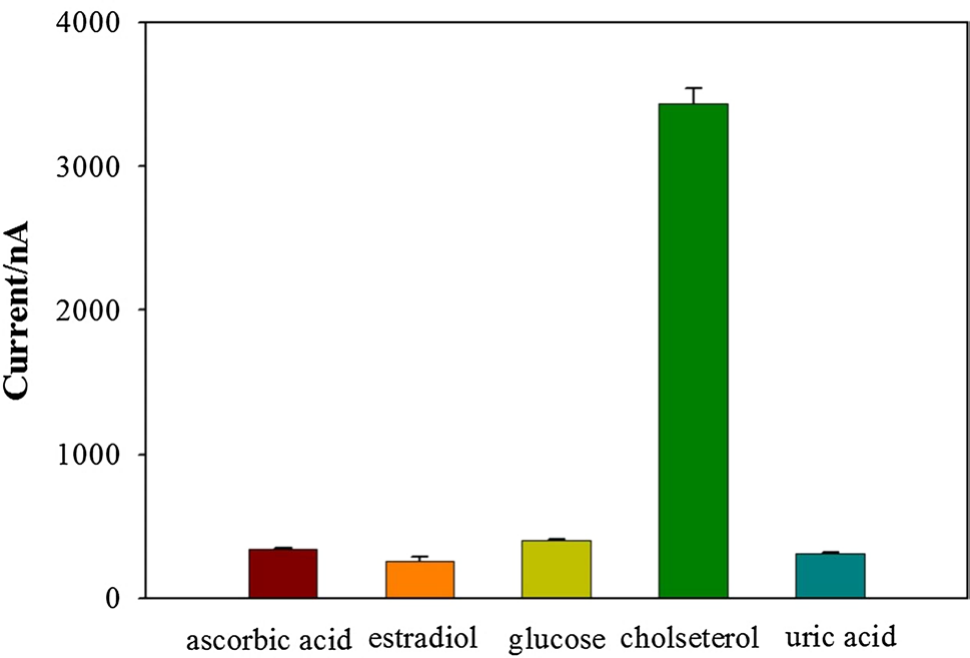
Current response of the biosensor toward cholesterol and interfering substances. The error bars are the standard error of the mean (*n* = 3 electrodes)

To evaluate the electrode selectivity, possible interfering substances, containing uric acid, estradiol, glucose, and ascorbic acid were investigated. The measurement was performed by adding substances (1.0 µM) instead of cholesterol. The interference effects of the above substances were negligible for cholesterol detection (Fig. 7). This indicated that the biosensor has excellent selectivity for cholesterol.

### Real-sample analysis

To assess the modified electrode application, total cholesterol in the serum samples was determined by the ChOx&ChEt/Au–Pt NPs/PAMAM–ZIF-67 biosensor. The dates shown in Table 2 indicated that cholesterol detection by the prepared electrode is consistent with the response signal.

**Table 2:** Measurement of cholesterol content in serum

Sample	Added (mM)	Found (mM)	Recovery (%)	RSD (%)
Human serum	1.504	1.532	101.86	2.66
	4.817	4.731	98.21	
	3.786	3.516	92.87	
	3.191	3.362	105.33	

## Conclusions

In this work, a kind of high-performance electrochemical biosensor consisting of PAMAM, ZIF-67, and bimetal nanoparticles was constructed. The biosensor showed high selectivity and a wide linear detection spectrum of 0.000015–10.24 mM. The minimum detection cholesterol content reached 3 nM. Furthermore, the biosensor displayed considerable value in detecting cholesterol during clinical diagnostic practice.

## Data Availability

Data are available on request.
